# Superior outcomes of total hip arthroplasty without prior lumbar arthrodesis: a systematic review and meta-analysis

**DOI:** 10.1007/s00590-023-03761-1

**Published:** 2023-10-17

**Authors:** Riccardo Giai Via, Filippo Migliorini, Francesco Bosco, Francesco Onorato, Davide Carlo Secco, Fortunato Giustra, Alessandro Dario Lavia, Matteo Giachino, Alessandro Massè

**Affiliations:** 1 Centro Traumatologico Ortopedico (CTO), Department of Orthopaedic Surgery, University of Turin, Via Gianfranco Zuretti, 29, 10126 Turin, Italy; 2 Department of Orthopaedic, Trauma and Reconstructive Surgery, RWTH University Medical Centre, 52074 Aachen, Germany; 3 Department of Orthopedics and Trauma Surgery, Academic Hospital of Bolzano (SABES-ASDAA), 39100 Bolzano, Italy; 4 Department of Orthopaedics and Traumatology, Ospedale San Giovanni Bosco di Torino - ASL Città di Torino, Turin, Italy; 5 Department of Economics, Boston College, Boston, MA USA; 6 Department of Orthopaedics and Traumatology (DiChirOnS), University of Palermo, Palermo, Italy

**Keywords:** Total hip arthroplasty, Hip replacement, Lumbar arthrodesis, Spinal fusion, Pelvic biomechanics, Complication, Dislocation

## Abstract

**Purpose:**

The number of patients undergoing total hip arthroplasty (THA) surgery after previous lumbar arthrodesis (LA) is rising. Literature suggests that LA may significantly impact pelvic biomechanics and potentially compromise the success of prosthetic hip replacement. This study aims to evaluate complication rates, dislocation rates, and revision rates in patients with prior LA undergoing THA surgery compared to those undergoing THA surgery without prior LA.

**Methods:**

A systematic review and meta-analysis were conducted following the Preferred Reporting Items for Systematic Reviews and Meta-Analyses (PRISMA) guidelines. A PICOS template was developed to ensure a structured approach. The search for relevant studies was performed across five databases, including Pubmed, Scopus, Embase, Medline, and Cochrane. The selected articles were evaluated based on the Levels of Evidence (LoE) criteria. The Coleman Methodology Score (mCMS) was employed to analyze the retrospective studies. This systematic review and meta-analysis were registered in the International Prospective Register of Systematic Reviews (PROSPERO). For the outcomes that allowed for a meta-analysis performed using R software, a *p* < 0.05 was considered statistically significant.

**Results:**

The final analysis included seventeen studies comprising a total of 3,139,164 cases of THA. Among these cases, 3,081,137 underwent THA surgery alone, while 58,027 patients underwent THA with a previous LA. The study investigated various factors, including dislocation rates, revision rates, and complication, as well as the surgical approach and type of implant used, for both the THA-only group and the group of patients who underwent THA with prior LA. The analysis revealed a statistically significant difference (*p* < 0.05) for all variables studied, favoring the group of patients who underwent THA alone without prior LA.

**Conclusions:**

This systematic review and meta-analysis demonstrated a statistically significant superiority in all analyzed outcomes for patients who underwent THA-only without prior LA. Specifically, patients with isolated THA implants experienced significantly lower incidences of THA dislocation, wound complications, periprosthetic joint infection, revision, and mechanical complications.

**Level of evidence:**

Level IV.

## Introduction

Total hip arthroplasty (THA) is a highly effective surgical procedure that has revolutionized the lives of millions of patients suffering from hip joint degeneration, osteoarthritis, or severe hip pain. It provides significant advancements in pain relief, functional outcomes, and overall quality of life. However, orthopedic surgeons face a unique challenge regarding a specific subset of patients requiring THA: those with a history of prior spinal arthrodesis.

The number of patients with a history of previous spinal stabilization undergoing total hip replacement (THR) surgery has been increasing substantially. This trend may be attributed to the widespread use of both techniques and the aging population. As a result, more and more patients are undergoing THR surgery after spinal arthrodesis. Some authors in Literature indicate a staggering 293% increase in patients undergoing both THR and spinal arthrodesis surgery over the past 10 years [1–3].

Patients undergoing spinal stabilization before THR surgery are at a higher risk of dislocation and subsequent revision than those without prior spinal arthrodesis, as reported in the Literature [2–9].

When the lumbar spine is fused through arthrodesis, known as lumbar arthrodesis (LA), the spine's ability to adjust and change lordosis during postural shifts is compromised. This limitation also affects the pelvis and its mechanisms for pelvic variation during postural adjustments mentioned earlier [10, 11]. Essentially, the patient's pelvis may be locked in two ways: either as if they are always standing or "stuck standing," resulting in reduced PT and reduced acetabular anteversion, or as if they are always sitting or "stuck sitting," leading to increased PT and acetabular anteversion [8].

Our body employs compensatory mechanisms by increasing femoral mobility for these biomechanical changes. However, this increased mobility increases the risk of anterior and posterior impingement, further elevating the risk of dislocation, especially during postural changes as described above [6–8, 12]. A comprehensive evaluation of the patient's spine is required to address the unique challenges posed by THA with prior LA. This evaluation involves thoroughly reviewing imaging studies, clinical examinations, and meticulous preoperative planning.

The purpose of this systematic review and meta-analysis is to highlight the revision, dislocation, periprosthetic joint infection (PJI) and aseptic loosening rates of patients undergoing THR surgery and compare them with those of patients undergoing THR surgery with previous LA to help orthopedic surgeons determine the optimal surgical approach, implant selection, and placement to ensure the stability, longevity, and functional success of the hip prosthesis.

## Material and methods

### Research question

The Preferred Reporting Items for Systematic Reviews and Meta-Analyses (PRISMA) flow diagram was used to perform the research and select the studies included in this systematic review [13–29]. Two independent authors (RGV and FB) searched and reviewed the final included articles to avoid possible bias. In case of discrepancy, a third author (FG) was consulted.

### Methodological quality assessment

Each included article was analyzed in our study according to the Oxford Centre for Evidence-Based Medicine 2011 Levels of Evidence (LoE). With this tool, articles were classified from 1 to 5, where LoE 1 represented a better design, methodological quality, and lower risk of bias in the study under review. The Coleman Methodology Score (mCMS), modified by Ramponi et al. [30], was used to analyze the retrospective studies. These tools were used by three authors (RGV, FO, DCS), and a fourth author (FB) was considered to resolve any uncertainties further. Statistical analysis was performed by a professional statistician (AL).

### Inclusion and exclusion criteria

The Patient, Intervention, Comparison, Outcomes, and Study (PICOS) design was used to classify and answer clinical questions according to the PRISMA checklist: patient (P), patients who sustained a total hip arthroplasty after LA; intervention (I) patients who underwent total hip replacement after LA; comparison (C), patients who underwent THR without previous LA; outcomes (O) clinical, radiographic outcomes and dislocation and revision rates; studies (S) retrospective studies. Inclusion criteria for the reviewed studies were articles about patients undergoing total hip replacement with prior LA compared to patients undergoing THR without prior LA surgery, written in English, studying human subjects, published between 2000 and 2023 with a minimum follow-up of 12 months, RCTs and retrospective studies with LoE 1 to 4. Biochemical and in vitro studies, case reports, editorials, book chapters, technical reports, pre-clinical studies, and review articles were excluded from the search. We also excluded studies with LoE 5 for a better-quality study.

### Study selection and search strategy

**Fig. 1:**
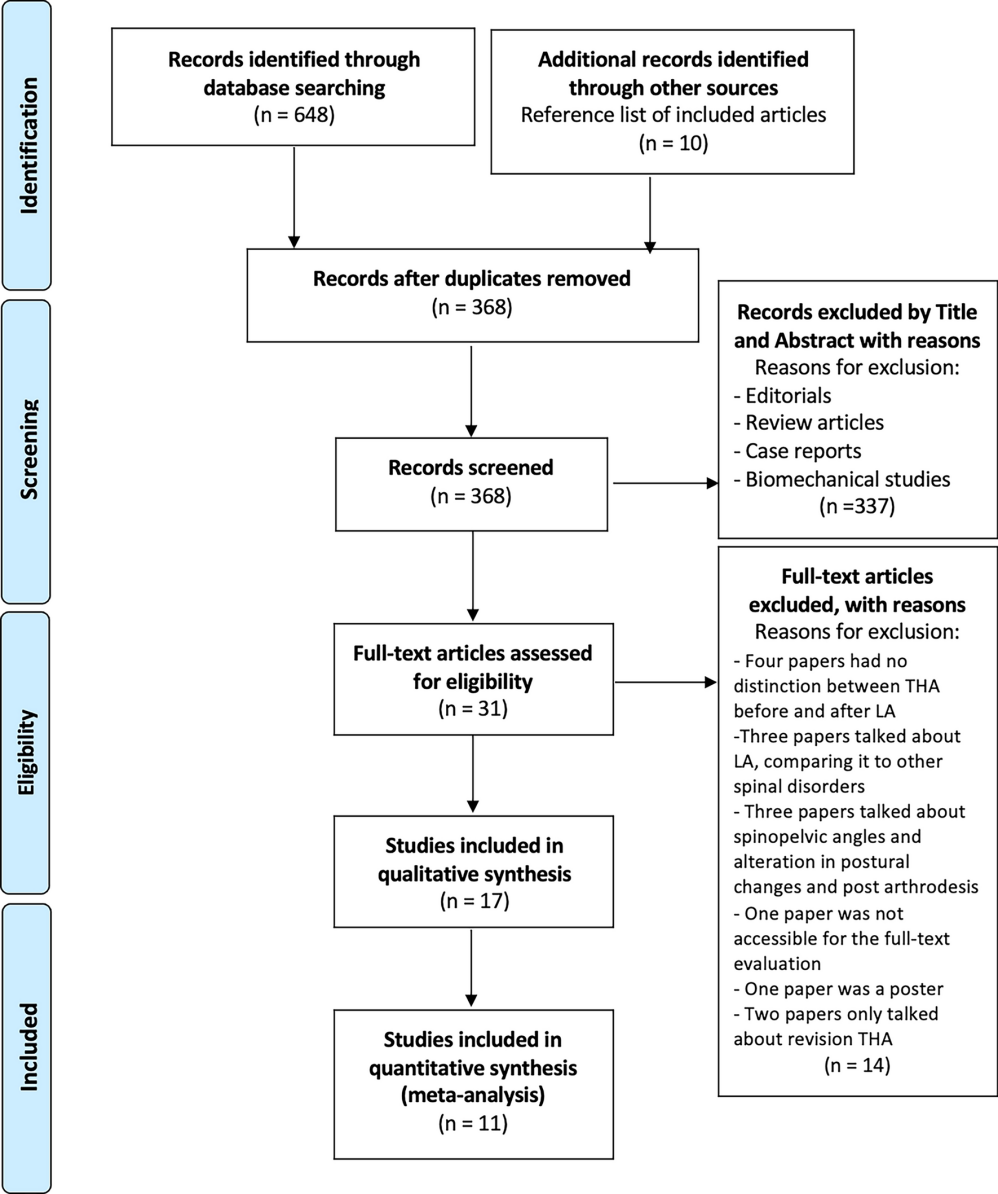
PRISMA flow diagram. *THA* total hip arthroplasty, *LA* lumbar arthrodesis. *CI* confidence interval, *OR* odds ratio

A comprehensive literature search was conducted in five databases (PubMed, Scopus, Embase, Cochrane databases, and MEDLINE) with the following MeSH terms: ((Total Hip arthroplast*) OR (THA) OR (Total hip replacement) OR (THR)) AND ((lumbar stabilization) OR (lumbar arthrodesis) OR (lumbar spine fusion)). With the above MeSH terms, we found a total of 658 studies. After the exclusion of duplicates, 368 studies were included. After reviewing the title and abstract of these studies, 331 studies were excluded resulting in 31 eligible studies. These studies underwent a thorough full-text evaluation to determine their eligibility, and based on the predetermined exclusion and inclusion criteria, a total of 17 studies [13–29] were deemed suitable for qualitative analysis. The selected studies specifically examined and compared complication and revision rates in patients who underwent THA with previous LA and those who only underwent THA. The PRISMA diagram illustrating the study selection process is presented in Fig. 1.

### Data extraction

The data extracted from the included articles were systematically recorded on a template, which comprised the following elements: author and publication year, study design, patient sample size, mean age of the participants, rates of complications and revision, and details regarding the surgical approach employed for total hip replacement. This comprehensive template allowed for the organization and analysis of key information. The template facilitated the capture of relevant data providing a more comprehensive understanding of the study findings.

### Statistical analysis

Statistical analyses were performed using R software, version 4.1.3 (2022; R Core Team, Vienna, Austria). A comprehensive meta-analysis was conducted on six variables: 90 day THA revision rate, 1 year THA revision rate, overall THA revision rate, overall THA PJI rate, overall THA dislocation rate, and overall THA aseptic loosening rate. In this analysis, a total of eleven studies were included [13, 15, 16, 21–24, 26–29], with each study providing data on one or more of the variables mentioned above. For each variable, the odds ratio (OR) was calculated to compare the occurrence of events between two groups: THA and THA with prior LA. The Mantel–Haenszel Method was applied to obtain a weighted estimate under a fixed-effects model. To assess heterogeneity among the studies, Cochran's Q test and Higgins' I^2 statistics were conducted. A *p* value of 0.05 was used as the threshold to determine the statistical significance of the odds ratio. Additionally, funnel plots and Egger's tests were performed to examine the possibility of publication bias.

## Results

A total of 3,139,164 THAs were considered in this comprehensive analysis, incorporating data from 17 studies [13–29]. Among these, 58,027 patients underwent THA after a previous LA, while 3,081,137 patients were treated with THA only. It is worth noting that four studies [13–16] contributed a disproportionately high number of patients, accounting for 50,025 THAs with LA and 2,756,489 THA-only cases. In terms of study design, all the included studies were retrospective. These articles were published within the last 6 years, from 2017 to 2022. Table 1 provides a concise summary of the main demographic characteristics of the study population.

**Table 1:** Main demographic characteristic of patients collected in the studies included in the systematic review and meta-analysis

Authors and year of publication	Study design	LOE	Sample size patients	Sample size patients	Sample size patients	Age			Gender THA		Gender THA + LA	Gender THA + LA	Follow-up
			Total	THA	THA + LA	Total	THA	THA + LA	M	F	M	F	Mean (range), years
			N	N	N	Mean, years	Mean, years	Mean, years	%	%	%	%	
Penrose et al. 2018 [13]	RS	III	881,266	862,627	18,639	/	/	/	38.18	60.7	34.01	65.06	Range 2–7
Salib et al. 2019 [19]	RS	III	291	194	97	71	71	71	44	56	44	56	6 (2–17)
Sing et al. 2016 [14]	RS	III	598,995	589,300	9,695	/	/	/	38.1	61.9	/	/	At least 2
Perfetti et al. 2017 [27]	RS	III	1,868	934	934	/	64.3	63.5	39.8	60.2	36.7	63.3	At least 1
Yang et al. 2022 [15]	RS	III	472,502	465,558	6,944	/	/	/	43.5	56.5	/	/	2
Huerfano et al. 2020 [20]	RS	III	27	17	10	/	37.5	70.6	29.4	70.6	30.0	70.0	At least 1
Buckland et al. 2017 [16]	RS	III	853,751	839,004	14,747	/	/	/	38.6	61.4	34.2	65.8	At least 1
Grammatopoulos et al. 2019 [21]	RS	III	107	60	47	68	69	67	30.0	70.0	29.7	70.3	3.7 (1–11)
Pollard et al. 2022 [28]	RS	III	14,545	14,217	328	/	/	/	33.5	66.5	36.9	63.11	at least 2
Di Martino et al. 2021 [24]	RS	III	68,598	67,919	679	66.4	68.8	66.1	39.1	60.9	38.7	61.3	7.1(0–18.0) *; 5.2(0–19.0) **
Mononen et al .2020 [22]	RS	III	101,443	100,528	915	67.5	67.9	67.1	44.2	55.8	32.3	67.7	9.9 ***; 8.8****
Goyal et al. 2022 [25]	RS	III	582	250	332	64	/	66.1	/	/	36.40	63.60	At least 1
Yang et al. 2020 [29]	RS	III	85,595	80,131	2637	/	/	/	/	/	/	/	At least 1
Malkani et al. 2018 [26]	RS	III	62,387	60,578	1809	/	/	/	/	/	/	/	At least 1
Nessler et al. 2020 [17]	RS	III	93	0	93	/	/	65.5			39.8	60.2	2.7 (1–10.3)
Barry et al. 2017 [23]	RS	III	105	70	35	/	68.4	68.5	44.3	55.7	40.0	60.0	At least 1
Chalmers et al. 2020 [18]	RS	III	86	0	86	/	/	/	/	/	19.0	71.0	3 (1–7)

*LoE* oxford centre for evidence-based medicine 2011 levels of evidence, *N* number of evaluation cases, *THA* total hip arthroplasty, *LA* lumbar arthrodesis, *y* years, *M* male, *F* female, *%* percentage, *RS* retrospective study, / not reported

^*^Only THA, **THA + LA, ***MoM group, ****non-MoM group

Five studies [13–15, 18, 19] reported their outcomes by distinguishing spinal fusion procedures based on the number of vertebral levels involved in the arthrodesis. As for the surgical techniques utilized in THA, considerable heterogeneity was observed, except for two studies [20, 21] that exclusively included patients treated with the posterolateral approach. For detailed information on surgical approaches and complications specific to each study, refer to Tables 2, 3. The analysis of revision rates at different time points consistently revealed an increased incidence of revision rate when THA was performed after LA, as presented in Table 4.

**Table 2:** Surgical approaches used in the included studies

Authors and year of publication	Surgical approach THA alone					Surgical approach THA + LA				
	Anterior	Antero lateral	Direct lateral	Posterolateral	Other	Anterior	Antero lateral	Direct lateral	Posterolateral	Other
	% (N)	% (N)	% (N)	% (N)	% (N)	% (N)	% (N)	% (N)	% (N)	% (N)
Salib et al. 2019 [19]	9 (18)	51 (98)	0 (0)	40 (78)	0 (0)	9 (9)	51 (49)	0 (0)	40 (39)	0 (0)
Huerfano et al. 2020 [20]	0 (0)	0 (0)	0 (0)	100.0	0 (0)	0 (0)	0 (0)	0 (0)	100.0	0 (0)
Grammatopoulos et al. 2019 [21]	0 (0)	0 (0)	0 (0)	100.0	0 (0)	0 (0)	0 (0)	0 (0)	100.0	0 (0)
Di Martino et al. 2021 [24]	6.9	/	59.5	32.8	0.8	7.9	/	61.3	29.3	1.5
Goyal et al. 2022 [25]	0 (0)	0 (0)	0 (0)	0 (0)	0 (0)	26.8 (89)	0 (0)	73.2 (243)	0 (0)	0 (0)
Nessler et al. 2020 [17]	0 (0)	0 (0)	0 (0)	0 (0)	0 (0)	11.9 (11)	/	16.1 (15)	67.7 (63)	4.3 (4)
Barry et al. 2017 [23]	8.6(6)	614(43)	0 (0)	30(21)	0 (0)	2.9 (1)	51.4 (18)	0 (0)	45.7 (16)	0 (0)

*THA* total hip arthroplasty, *LA* lumbar arthrodesis, *N* number % percentage, / not reported

**Table 3:** Complications, THA alone vs THA + LA

Complication	Authors and publication year	Dual mobility cup, %	THA alone incidence	THA + LA incidence	*P* value
90 days hip dislocation	Penrose et al. 2018 [13]	/	1.5%	3.1%	*p* < 0.001
	Perfetti et al. 2017 [27]	/	0.3%	1.4%	*p* = 0.12
	Barry et al. 2017 [23]	/	0%	2.9%	*p* > 0.001
	Nessler et al. 2020 [17]	100%	/	0%	/
1 year hip dislocation	Penrose et al. 2018 [13]	/	2.2%	4.3%	*p* < 0.001
	Sing et al. 2016 [14]	/	2.0%	4.2%	*p* < 0.001
	Perfetti et al. 2017 [27]	/	0.4%	3.0%	*p* < 0.001
	Yang et al. 2022 [15]	0%	1.9%	3.8%	*p* < 0.001
	Huerfano et al. 2020 [20]	0%	0%	20.0%	*p* > 0.001
	Buckland et al .2017 [16]	/	1.5%	3.1%	*p* < 0.001
	Pollard et al. 2022 [28]	/	3.9%	8.5%	*p* < 0.001
	Nessler et al. 2020 [17]	100%	/	0%	/
Overall hip dislocation	Penrose et al. 2018 [13]	/	3.0%	5.5%	*p* < 0.001
	Grammatopoulos et al. 2019 [21]	/	3.3%	2.1%	/
	Di Martino et al. 2021 [24]	/	0.8%	2.1%	*p* > 0.001
	Mononen et al .2020 [22]	0.0003%	2.8%	4.7%	*p* < 0.001
	Goyal et al. 2022 [25]	0%	/	0.9%	/
	Yang et al. 2020 [15]	/	2.0%	3.3%	*p* < 0.001
	Malkani et al. 2018 [26]	/	4.8%	7.4%	*p* < 0.001
	Nessler et al. 2020 [17]	100%	/	0%	/
	Chalmers et al. 2020 [18]	100%	/	0%	/
Overall aseptic loosening	Penrose et al. 2018 [13]	/	2.3%	3.1%	*p* < 0.001
	Di Martino et al. 2021 [24]	/	1.8%	1.3%	*p* > 0.001
	Barry et al. 2017 [23]	/	1.4%	0%	*p* > 0.001
	Nessler et al. 2020 [17]	100%	/	1.1%	/
Superficial wound infections	Penrose et al. 2018 [13]	/	0.8%	1.3%	*p* < 0.001
	Salib et al. 2019 [19]	0%	0%	5.0%	/
	Yang et al. 2020 [15]	/	1.9%	2.4%	*p* = 0.007
	Barry et al. 2017 [23]	/	2.8%	2.8%	/
	Chalmers 2020 [18]	100%	/	1.3%	/
Overall PJI	Penrose et al. 2018 [13]	/	2.3%	3.2%	*p* < 0.001
	Grammatopoulos et al. 2019 [21]	/	0%	8.5%	*p* = 0.080
	Yang et al. 2020 [15]	/	2.3%	3.1%	*p* < 0.001
	Nessler et al. 2020 [17]	100%	/	0%	/
	Chalmers et al. 2020 [18]	100%	/	1.3%	/

*PJI* prosthetic joint infection, *THA* total hip arthroplasty, *LA* lumbar arthrodesis, /: not reported

**Table 4:** Revision rate, THA alone vs THA + LA

Revision rate	Authors and year of publication	THA alone incidence	THA + LA incidence	*P* value
90 day	Penrose et al. 2018 [13]	1.8%	2.7%	*p *< 0.001
	Perfetti et al. 2017 [27]	0.5%	1.9%	*p* = 0.060
	Barry et al. 2017 [23]	2.9%	14.3%	*p* = 0.040
1 year	Penrose et al. 2018 [13]	2.9%	4.9%	*p* < 0.001
	Perfetti et al. 2017 [27]	0.9%	3.9%	*p* < 0.001
	Yang et al. 2022 [29]	2.5%	4.9%	*p* < 0.001
	Buckland et al. 2017 [16]	0.2%	0.4%	*p* < 0.001
Overall	Penrose et al. 2018 [13]	4.8%	7.0%	*p* < 0.001
	Grammatopoulos et al. 2019 [21]	0%	8.5%	*p* = 0.020
	Pollard et al. 2022 [28]	4.3%	9.4%	*p* < 0.001
	Di Martino et al. 2021 [24]	3.1%	3.2%	*p* = 0.024
	Mononen et al .2020 [22]	8.7%	12.0%	/
	Yang et al. 2020 [15]	3.8%	7.8%	*p* < 0.001
	Malkani et al. 2018 [26]	4.6%	6.9%	*p* < 0.001
	Sing et al. 2016 [14]	3.4%	6.1%	*p* < 0.001
	Chalmers et al. 2020 [18]	/	4.0%	/

*THA* total hip arthroplasty, *LA* lumbar arthrodesis, / not reported

**Fig. 2:**
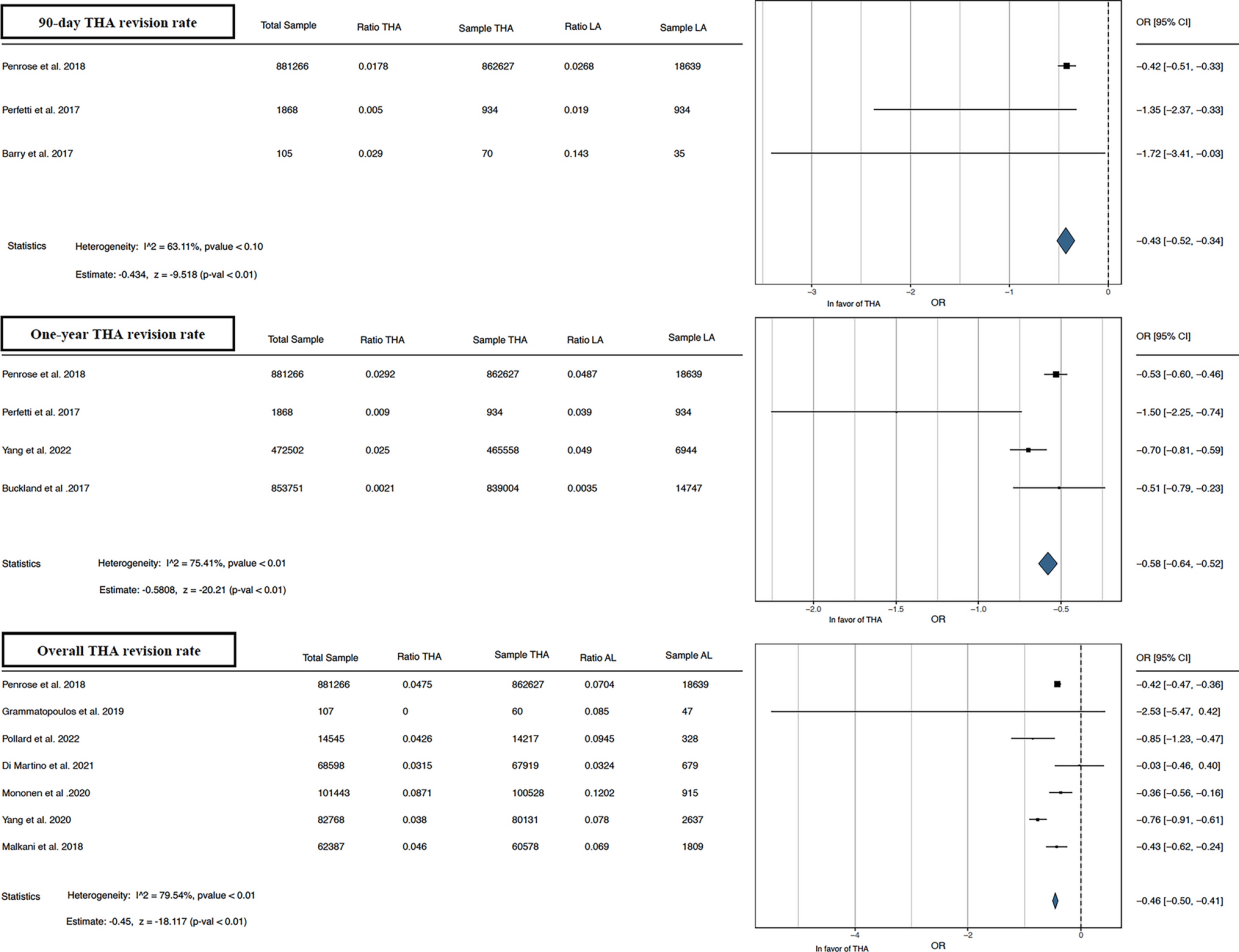
90 day, 1 year, and overall THA revision rate. *THA* total hip arthroplasty, *LA* lumbar arthrodesis. *CI* confidence interval, *OR* odds ratio

**Fig. 3:**
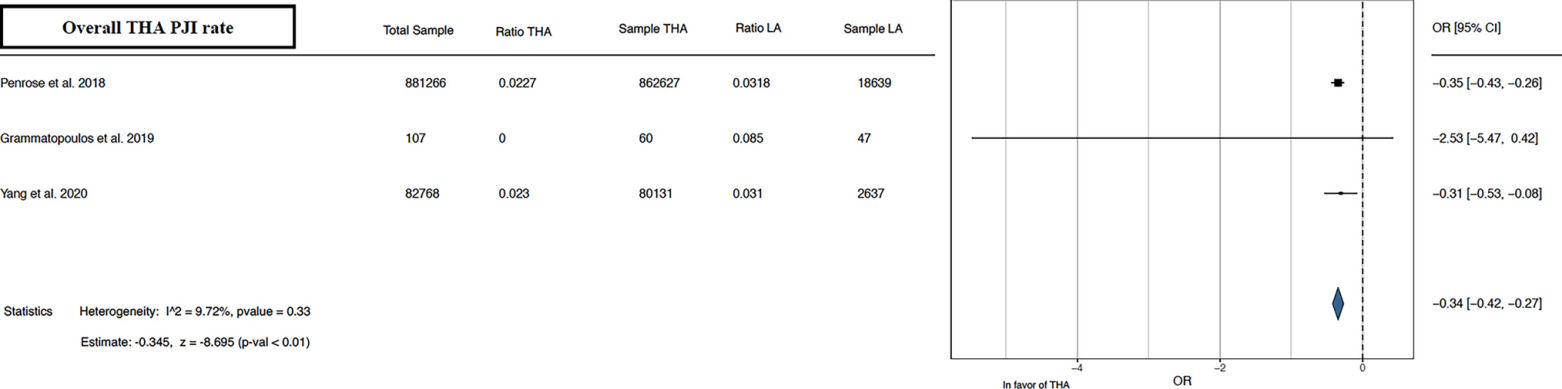
Overall THA PJI rate. *THA* total hip arthroplasty, *PJI* periprosthetic joint infection, *LA* lumbar arthrodesis. *CI* confidence interval, *OR* odds ratio

**Fig. 4:**
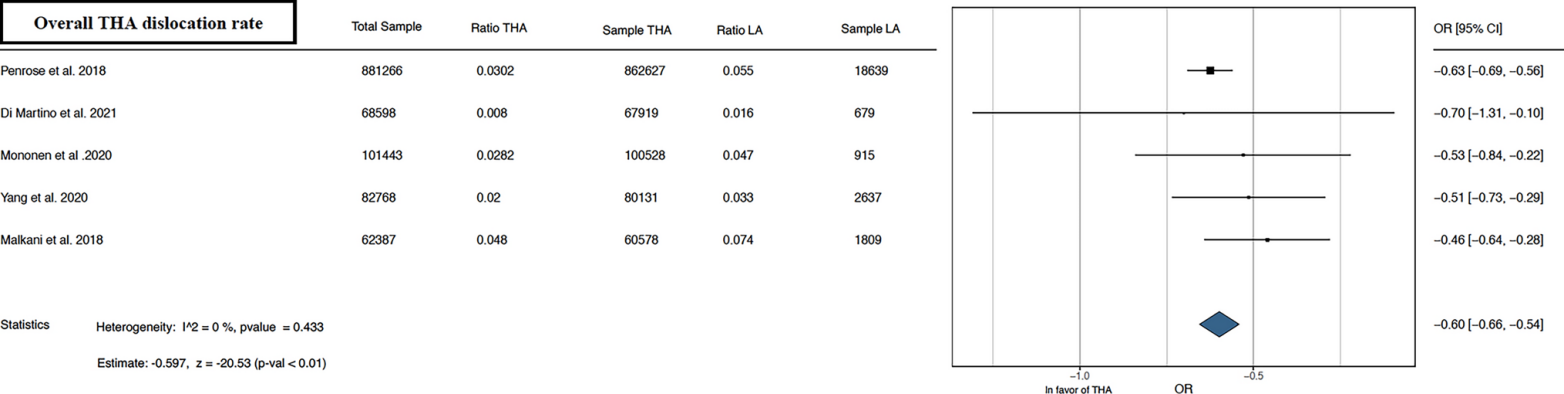
Overall THA dislocation rate. *THA* total hip arthroplasty, *LA* lumbar arthrodesis. *CI* confidence interval, *OR* odds ratio

**Fig. 5:**
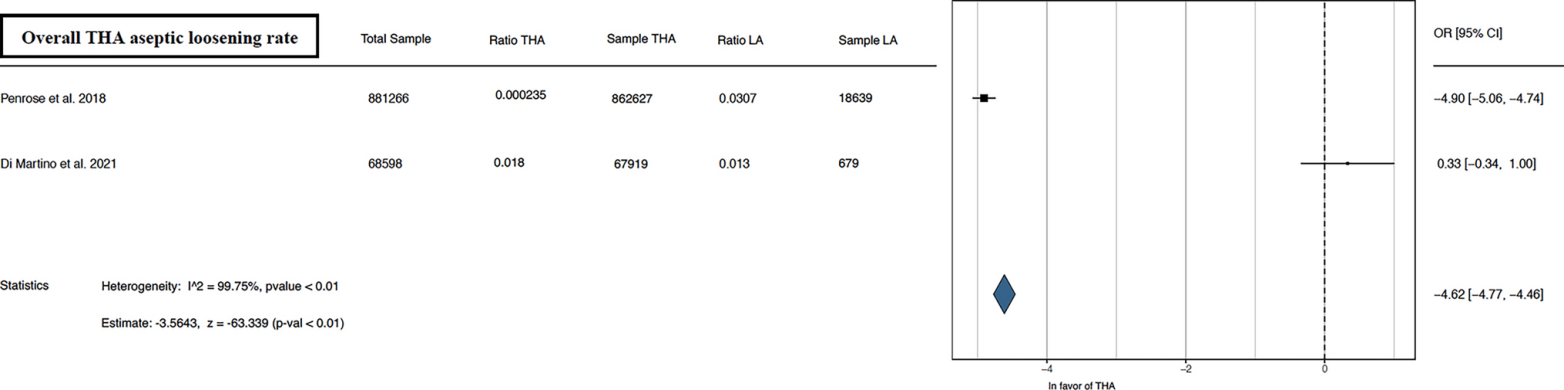
Overall THA aseptic loosening rate. *THA* total hip arthroplasty, *LA* lumbar arthrodesis. *CI* confidence interval, *OR* odds ratio

The meta-analysis conducted demonstrated better results in the outcomes analyzed for THA alone than for THA after LA. Forest plots illustrating the 90 day, 1 year, and overall THA revision rate [13, 15, 16, 21–24, 26–29] favored THA alone, as depicted in Fig. 2. Furthermore, forest plots of overall THA PJI rate [13, 21, 29] and overall THA dislocation rate [13, 22, 24, 26, 29] favored THA alone, as shown in Figs. 3, 4. Lastly, concerning the overall THA aseptic loosening rate, one study significantly favored THA alone [13], while another favored THA with LA [24]. Nevertheless, the comprehensive analysis revealed a significant advantage for THA alone, as shown in Fig. 5.

## Discussion

The most important finding of this study was the presence of superior outcomes in patients who underwent THA compared to patients who received a THA after a previous LA. Specifically, patients with isolated THA implants observed a significantly lower incidence of THA dislocation, wound complications, PJI, revision, and mechanical complications.

Onggo et al. [12] conducted a meta-analysis and systematic review, including ten studies (28,396 versus 1,578,687 with and without LA, respectively). PROMs were lower, and the rate of dislocations, revisions, and all other complications were 5.4, 6.3, and 4.6, higher in the LA group, respectively [12]. Recently, the same authors conducted another systematic review, comparing patients who received the LA either before (*N* = 43,880) or after (*N* = 25,558) THA [31]. The analysis did not find a statistically significant difference in the rate of [31], suggesting that the timing of LA could be an independent risk factor for complications. An et al. [32] investigated the impact of LA on patients undergoing THA in six studies (1,456,898 patients), showing a lower functional outcome for the LA group, along with a twofold higher risk of dislocation and a threefold higher risk of revision surgery [32].

**Fig. 6:**
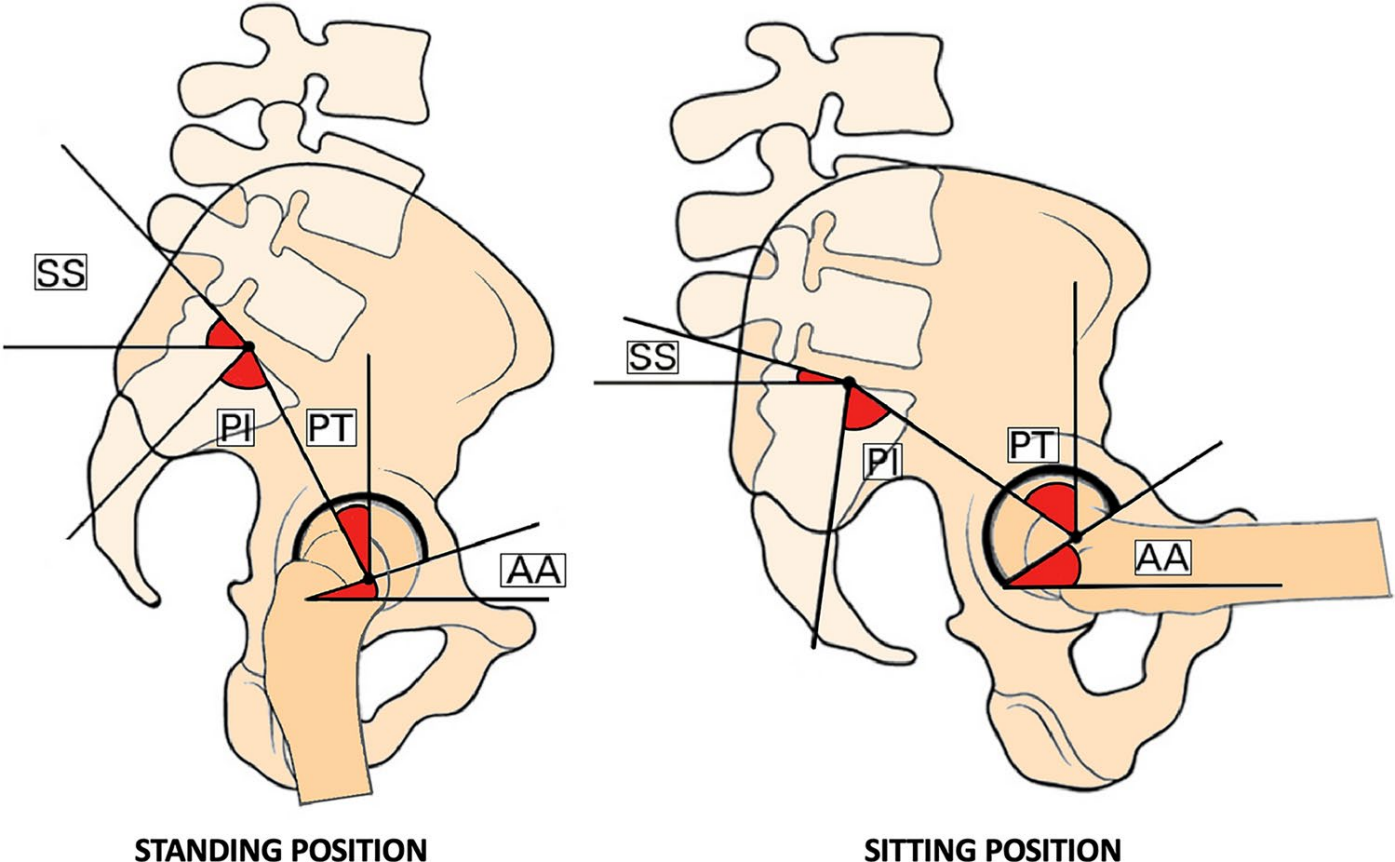
This image illustrates the natural variation in angles between a typical spine's standing and sitting positions. On the left, you can observe a standard standing posture characterized by an anteverted pelvis with a low PT, a high SS, and minimal AA. On the right, the image portrays a retroverted pelvis with a higher PT, increased AA, and a reduced SS. *PT* pelvic tilt, *SS* sacral slope, *AA* acetabular anteversion, *PI* pelvic incidence

**Fig. 7:**
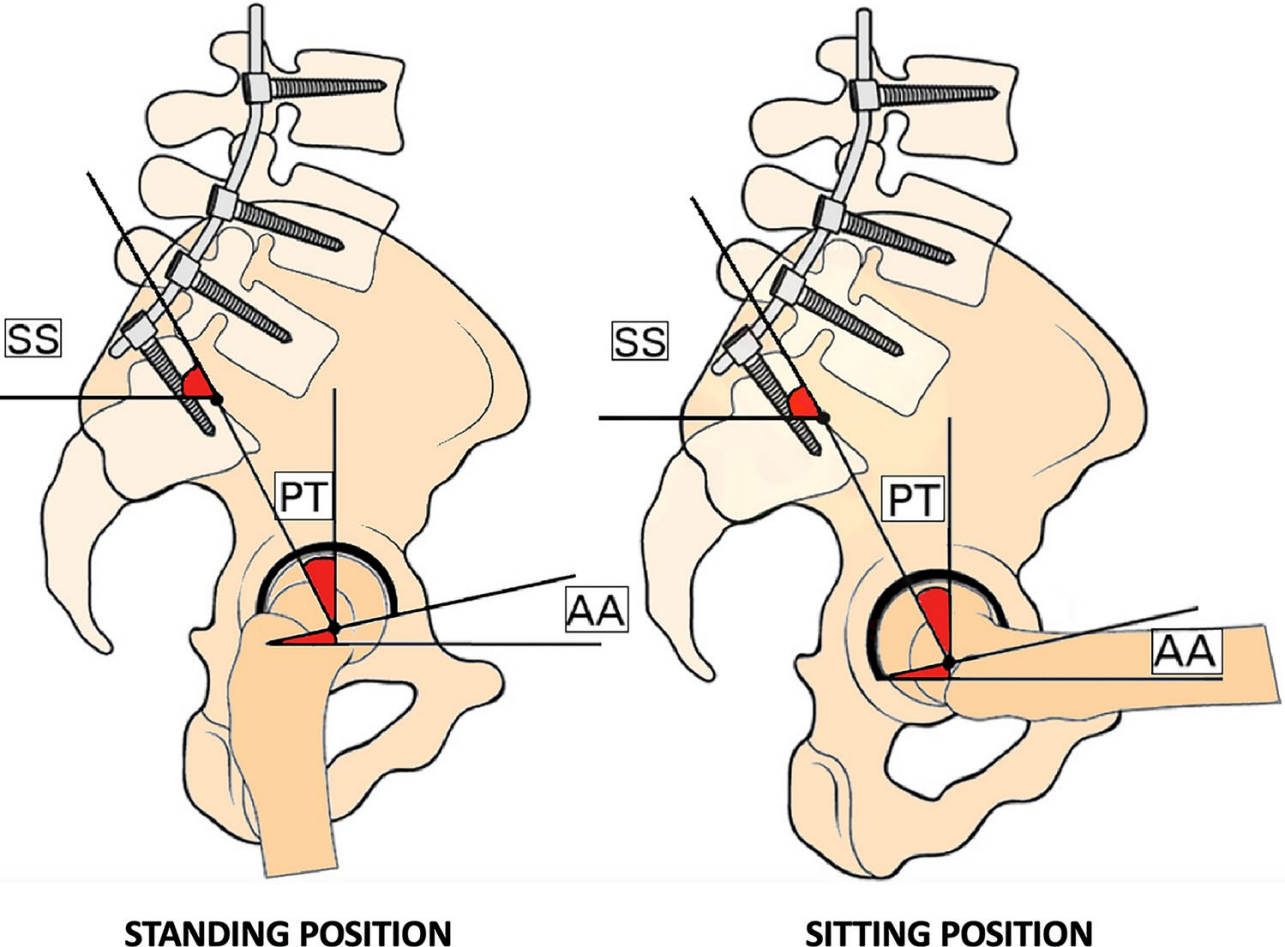
This image demonstrates how normal biomechanics are affected following LA. Notably, there is no significant alteration in the standard angles during the postural change. The pelvis remains immobilized, and both the PT and SS stay consistent, as does the AA. *LA* lumbar arthrodesis, *PT* pelvic tilt, *SS* sacral slope, *AA* acetabular anteversion

The reason for the higher rate of complication in patients undergoing LA before THR surgery is likely due to biomechanical changes caused by vertebral arthrodesis, specifically resulting in reduced pelvic tilt (PT) [8, 33, 34]. PT is the angle formed between a line connecting the femoral head's center to the sacral endplate's midpoint and a line starting from the center of the femoral head and perpendicular to the ground. The PT is closely related to pelvic incidence (PI), which represents a constant ratio inversely proportional to sacral slope (SS), and it means the angle between a line drawn from the midpoint of the sacral endplate to the center of the femoral heads and a perpendicular line dropped from the midpoint of the sacral endplate to the sagittal plane. The SS is the angle formed between a line drawn from the midpoint of the sacral endplate to the center of the femoral head and a horizontal line. In the standing position, PT decreases as SS increases. However, in the sitting position, PT is greater than SS [5]. During the transition from standing to sitting, there is a consistent increase in acetabular anteversion. Specifically, for every 1° of retroversion (increase in PT), there is a 0.7° increase in acetabular anteversion [6–9]. Normal biomechanics are impacted following LA, and the natural variation in angles between a typical spine's standing and sitting positions is not observed in patients treated with a previous LA (Figs. 6, 7).

The motion of the spine, pelvis, and hip determines the functional position of the acetabulum. The anterior and posterior pelvic tilting regulated the spinal configuration, which is essential to maintain the center of mass of the head and trunk directly above the legs and the position of the acetabulum over the femoral head [35, 36]. During staying, the hip extends, the pelvis tilts anteriorly, the spine becomes more lordotic, and the acetabulum relatively comes closer to the femoral head [12, 37]. During sitting, the hip flexion is associated with a posterior tilt of the acetabulum an average of 15°–20°. The spine becomes less lordotic, which allows the acetabulum to open an average of 15°–20° for the clearance of the hip [36, 38–40]. The patterns of imbalanced spinopelvic mobility due to LA are stuck standing and sitting [40]. The stuck standing pattern represents the combination of excess anterior pelvic tilting and hyper-lordosis of the lumbar spine when sitting [40]. Therefore, these patients have an increased risk of anterior impingement, leading to a possible posterior dislocation of the femoral head during hip flexion [40]. On the other hand, the stuck sitting pattern refers to excess posterior pelvic tilting and hypo-lordosis of the lumbar spine during standing [12, 40].

Patients with LA who underwent THA have a greater risk of posterior impingement and a greater rate of anterior hip dislocation during hip extension [40]. The fixation of a segment could lead to a hypolordic spine, resulting in a stuck sitting phenomenon [41]. The spinopelvic and hip joints act like two complementary hinges; therefore, every reduction or augmentation of the proper range of motion is compensated by the counterpart [42]. Subsequently, a decrease of 1° in the spinopelvic movement is related to an increase of 0.9° in the femoral motion [42]. The patients affected by the stuck sitting phenomenon have a compensatory increase in hip-femoral extension during functional and postural activities with an increased risk of posterior impingement; subsequently, anterior hip dislocation might occur [40, 43].

Dislocation is one of the most common complications and may lead to an unstable implant, which indicates revision [44, 45]. However, the cause of dislocation must be investigated. Indeed, several factors may promote THA dislocation, including osteophytes, enormous scar tissue, and suboptimal positioning of prosthetic elements, causing the anterior or posterior dislocation of the femoral head [46, 47]. The prosthetic head has a minor superior coverage for a higher inclination than 60°, while a low inclination below 30° can result in lateral impingement in abduction and flexion [12, 48, 49].

The cup positioning is crucial in preventing hip dislocation in LA patients [27, 32]. Historically, Lawinnek et al. [50] asserted that the "safe zone" for THA consists of 15° ± 10° of anteversion and 40° ± 10° of inclination [9, 50, 51].

The transverse acetabular ligament has been used as a reference during surgery to guide cup implantation within the safe zone described by Lawinnek et al. [50]. However, when there is an altered pelvic tilt, relying solely on the transverse acetabular ligament becomes less reliable, as each degree of posterior pelvic tilt corresponds to 0.7° of cup anteversion, leading to inaccurate cup positioning [52, 53].

Furthermore, the femur anteversion is essential in THA stability and preventing impingement [54, 55]. The anteverted acetabulum is in LA patients due to spinopelvic stiffness and hypo-lordosis of the spine, leading to loss of anterior pelvic tilting. Dandachli et al. [56] estimated that pelvic tilt changed the acetabular version with a decrease in anteversion ranging from 2.5° to 5° for every 5° of forward tilt. Therefore, the loss of pelvic tilt in patients with LA can be compensated with a minor femoral anteversion to obtain the hip anteversion in the target range [54, 57].

During THA, the acetabular cup and stem position must be customized to specific patient anatomy and biomechanics. More detailed and dynamic preoperative studies might help in preventing dislocations. In addition, patient-specific instrumentation has recently been developed to achieve a more precise acetabular cup position [58–60].

The strength of this meta-analysis is that the inclusive analysis of several studies that evaluated different postoperative variables comparing outcomes between patients undergoing THA alone and those undergoing THA after LA yielded robust results. Appropriate statistical methods were used to assess heterogeneity and publication bias. This study provides important clinical insights to guide the management of patients undergoing THA and LA surgery.

The limitations of this study include the restricted number of included studies, the heterogeneity among the analyzed studies, the possible publication bias, the presence of inconclusive results in some studies, the dependence on the data available in publications, the possible presence of confounding factors that were not considered, the use of only one statistical software for the analysis, and the possible limited generalizability of the results due to the specificities of the targeted populations and procedures. Furthermore, heterogeneity and potential bias could affect the validity and reliability of the study conclusions. Therefore, it is essential to interpret the results cautiously and consider further research to confirm the results obtained.

## Conclusions

This systematic review and meta-analysis provided compelling evidence of a statistically significant superiority in various outcome measures for patients who underwent THA-only without prior LA. These findings suggest that prior LA is associated with worse outcomes, highlighting the need for further high-quality studies, including randomized clinical trials. These studies would contribute to clarifying the most appropriate treatment solutions, such as the optimal surgical approach, dual mobility implants, or implants with larger heads, to mitigate the high rates of complications observed in patients with prior LA.

## Data Availability

Dataset analysed in this study is available from the corresponding author on reasonable request.
